# Dicer and miRNA in relation to clinicopathological variables in colorectal cancer patients

**DOI:** 10.1186/1471-2407-11-345

**Published:** 2011-08-10

**Authors:** Johannes Stratmann, Chao-Jie Wang, Sebastian Gnosa, Åsa Wallin, David Hinselwood, Xiao-Feng Sun, Hong Zhang

**Affiliations:** 1 Division of Biomedicine, Systems Biology Research Centre, School of Life Sciences, Skövde University, SE-541 28, Skövde, Sweden; 2 Division of Oncology, Department of Clinical and Experimental Medicine, Faculty of Health Sciences, Linköping University, SE-581 85, Linköping, Sweden

**Keywords:** CRC, Dicer, miRNAs, Prognosis, qPCR

## Abstract

**Background:**

Dicer is aberrantly expressed in several types of cancers. Applying real-time PCR, we detected the expression of Dicer mRNA in normal mucosa (n = 162), primary colorectal cancer (CRC) (n = 162) and liver metastasis (n = 37), and analysed the relationship between Dicer expression and clinicopathological features. We also correlated the expression of Dicer mRNA to the miRNA expression of miR-141, miR-200a, miR-200b, mir-200c and miR-429 in liver metastases.

**Methods:**

RT-PCR and qPCR were used to analyse the Dicer expression in normal mucosa, primary tumour and liver metastasis by using the High Capacity cDNA Reverse Transcription Kit and TaqMan™^® ^Gene Expression assays for *Dicer *and *GAPDH*. RT-PCR and qPCR were used to detect miRNA expression in liver metastases by utilizing TaqMan^® ^MicroRNA Reverse Transcription Kit and TaqMan^® ^miRNA Assays. Statistical analyses were performed with STATISTICA.

**Results:**

Dicer expression in rectal cancer (3.146 ± 0.953) was higher than in colon cancer (2.703 ± 1.204, P = 0.018). Furthermore the Dicer expression was increased in primary tumours (3.146 ± 0.952) in comparison to that in normal mucosa from rectal cancer patients (2.816 ± 1.009, P = 0.034) but this is not evident in colon cancer patients. Dicer expression in liver metastases was decreased in comparison to that of either normal mucosa or primary tumour in both colon and rectal cancers (P < 0.05). Patients with a high Dicer expression in normal mucosa had a worse prognosis compared to those with a low Dicer expression, independently of gender, age, tumour site, stage and differentiation (P < 0.001, RR 3.682, 95% CI 1.749 - 7.750). In liver metastases, Dicer was positively related to miR-141 (R = 0.419, P = 0.015).

**Conclusion:**

Dicer is up-regulated in the early development of rectal cancers. An increased expression of Dicer mRNA in normal mucosa from CRC patients is significantly related to poor survival independently of gender, age, tumour site, stage and differentiation.

## Background

CRC is one of the world's leading cancer diseases and third leading cause of cancer death world-wide [1]. Only about 5-10% of the CRC cases arise on a defined hereditary background due to two main diseases, familial adenomatous polyposis (APC) and hereditary nonpolyposis colorectal cancer [2]. Most of the cases seem to occur sporadically without a hereditary background, and are related to environmental factors (e.g., red meat, high-fat diet, inadequate intake of fibre, obesity, sedentary lifestyle, diabetes mellitus, smoking, high consumption of alcohol) [3] and also to inflammatory bowel diseases [4,5].

Recently it has been speculated that microRNAs (miRNAs) play a major role in cancer development [6]. MicroRNAs are small non-coding RNAs with a length of approximately 22 nucleotides. These are known to work as switches for genes and their correlated proteins, have hundreds of targets in tumour suppressor or oncogene pathways [6] and are often referred to as "oncomirs" [7]. More specifically the miRNAs of the miR-200 family have been shown to control epithelial to mesenchymal transition (EMT) by down-regulating the expression of the Zeb factors and controlling the metastatic ability of cancer cells [8].

One of the key enzymes in the miRNA generating process, Dicer [9], is a protein with a size of 219 kDa [10] and consists of two RNase III domains, RNase IIIa and RNase IIIb, which are responsible for the biogenesis of short interfering RNAs and miRNAs [11]. Dicer is located in the cytoplasm and associates there with miRNA precursors, the pre-miRNAs, resulting in cleavage to mature ~22 nucleotide miRNA-miRNA duplexes. Translational repression or degradation of mRNA occurs when miRNA binds to the RNA induced silencing complex [12].

Studies on non-small cell lung carcinoma (NSCLC) show that Dicer protein is down-regulated in areas of invasion and advanced carcinomas [13], and furthermore that reduced mRNA expression is significantly associated with poor patient survival [14]. Similar results are obtained in studies on Dicer mRNA in breast cancers [15], as well as in ovarian cancer patients where Dicer mRNA was shown to be decreased in ovarian cancers in comparison to benign and normal samples [16], furthermore, low Dicer protein expression is significantly associated with advanced tumour stages and with decreased survival [12]. In contrast, another study on ovarian cancer patients showed that Dicer mRNA and protein expression are significantly up-regulated in cancers, and increased Dicer is related to reduced disease-free survival [17]. A study on prostate cancers exhibits up-regulated Dicer in 81% of prostate cancers, and increased Dicer expression is related to advanced stages [18]. Recently, a study on CRC cancer also shows that high Dicer expression is related to poor patient survival [19]. These studies show that there are differentiated patterns and roles of Dicer expression in different types of tumours.

Major aims of our study were to determine the Dicer mRNA level in the normal mucosa and primary tumours from CRC patients as well as liver metastases from independent CRC patients. Further, we examined whether the Dicer mRNA level in these different tissues was related to the patient survival as well as to other clinicopathological variables. Dicer is directly connected with miRNAs [9], who's up- or down regulations are strongly associated with the development of CRC [20], while a study in mice revealed that certain miRNAs can be generated independent from Dicer and require catalysis by the Argonaute protein [21]. More specifically, it was shown that high expression of miR-200c in CRC patients was significantly associated with poor patient survival [22]. Therefore, we decided to analyse the miRNAs of the miR-200 family, miR-141, miR-200a, miR-200b, miR-200c and miR-429 in liver metastases since it is known that the members of the miR-200 family are functional linked to EMT, and may be involved in facilitating the metastatic behaviour of cancer cells [23]. Furthermore we examined the relationship of Dicer to the miRNA level in order to have an insight into their interaction. We report here, for the first time, that an increased Dicer mRNA level in normal mucosa from CRC patients is associated with a worse survival. Dicer is up-regulated in rectal cancers in comparison to colon cancers. Furthermore we found a correlation between Dicer and miR-141 expression in liver metastases.

## Methods

### Patients

The patient material included primary tumour, the corresponding normal mucosa and liver metastases collected at Linköping University Hospital between 1980 and 2009. Primary tumour and the corresponding normal mucosa (taken from uninvolved morphologically normal colorectal tissues) were obtained from 162 patients with primary CRC, whereas liver metastases were collected from 37 independent CRC patients. For each primary tumour patient, the primary tumour and the corresponding normal mucosa for comparison were collected. Median age of the CRC patients was 74 years (ranging from 35 to 94 years). Thirty-two out of 108 reported CRC patients were pre-treated with either pre-chemotherapy or pre-radiotherapy. Median age of the patients with liver metastases was 65 years (ranging from 37 years to 86 years). Twenty-six patients with liver metastases underwent pre-surgical chemotherapy. All specimens were flash-frozen in liquid nitrogen and then stored at -80°C. According to the histological diagnose criteria of the World Health Organization; all samples were examined by the pathologists at the Department of Pathology in Linköping University to confirm their histopathological type, TNM stage and metastasis. The survival analyses were based on overall survival.

### RNA extraction and RT-PCR

Total RNA was extracted from nitrogen flash frozen tissue using the TRizol reagent (Sigma-Aldrich, St. Louis, MO) and RNeasy Kit (75144, QIAGEN, Venlo, NL) according to the manufacturer's instructions. The concentration and integrity of RNA were measured with the Agilent 2100 Bioanalyzer (Agilent Technologies, Santa Clara, CA). For the RT-PCR the High Capacity cDNA Reverse Transcription Kit (Applied Biosystems, Foster City, CA) was utilized. 10 μL total RNA was reverse transcribed using MultiScribe™ Reverse Transcriptase according to the manufacturer's instructions, without an RNase inhibitor in a final volume of 20 μL. The program is the following: 25°C 10 min, 37°C 120 min, 85°C 5 min.

For miRNA analysis, total RNA inclusive of the small RNA fraction was extracted by using the mirVana miRNA Isolation Kit (Ambion Inc.; Austin TX), according to the manufacturers' protocol. For the RT-PCR the TaqMan^® ^MicroRNA Reverse Transcription Kit was used according to the manufacturer's protocol. The program was the following: 16°C 30 min, 42°C 30 min, 85°C 5 min.

### qPCR

The relative expression levels of Dicer were determined by qPCR with TaqMan™^® ^Gene Expression Fast Master Mix in Applied Biosystems 7900HT Fast Real-Time PCR System and normalised to *GAPDH*. Primers and hydrolysis probes were TaqMan™^® ^Gene Expression assays on demand for *Dicer *(Hs00229023_m1*) and *GAPDH *(4352934E) (Applied Biosystems). All the samples were performed in triplicates. The PCR amplification program was the following: 95°C 20 sec, 40 cycles of 95°C 1 sec and 60°C 20 sec. In addition, ddH_2_O as the non-template control was analysed for every plate.

qPCR on miRNAs was performed according to the manual from a recent miRNA study [24] with TaqMan^® ^Universal PCR Master Mix II and TaqMan miRNA Assays for mature miRNAs (Applied Biosystems) for hsa-mir-141 (Assay ID; 000463), -200a (Assay ID; 000502), -200b (Assay ID; 002251), -200c (Assay ID; 002300) and -429 (Assay ID; 001024). All samples were analysed in triplicates and RNU48 (Assay ID; 001006 ) was used as a reference gene. We followed the manual provided by Applied Biosystems (Amplification Efficiency of TaqMan^® ^Gene Expression Assays) to work on the samples, namely, for each gene of interest and reference gene the amplification efficiency was directly determined by the slope on the SDS-program provided by Applied Biosystems. The slope must be ≤ -3, 321 to get amplification efficiencies ≤ 100%. The calculation was done with the formula - 1 where E = amplification efficiency. For each real-time-PCR run, the slope was adjusted for each sample in order to assure that the amplification efficiencies were similar for each gene and each run. The data obtained from the qPCR was analysed by the ΔΔCt-method. The Dicer expression level was divided at a cut-off point of 75%, to distinguish between a low and high expression.

### Statistical Analysis

For all statistical analyses the program STATISTICA (StatSoft, Tulsa, OK) was utilized. The values for the mRNA level from the real-time PCR were transformed to log_2 _values and the data were normally distributed. Student's t-test was used to examine the differences in Dicer mRNA levels between the samples. Student's t-test or one-way ANOVA method was applied to analyse the relationships with clinicopathological variables. Kaplan-Meier method was used to calculate survival curves. Cox's Proportional Hazard Model was used to estimate the relationship between mRNA expression value and patients survival in univariate and multivariate analyses. Pearson correlation was used to analyse the relationship between Dicer and miRNAs. P-values of less than 0.05 were considered statistically significant.

## Results

### Dicer mRNA level in normal mucosa, primary tumour and liver metastasis

**Figure 1:**
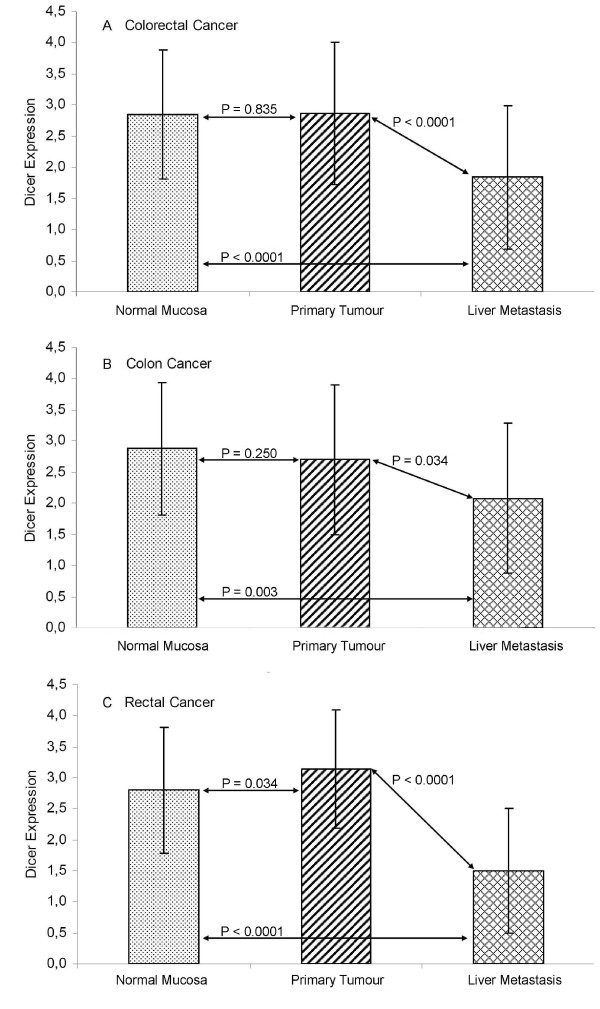
**The mRNA level of Dicer in normal mucosa, primary tumour and liver metastasis**. **(A) **Dicer mRNA level in normal mucosa, primary tumour and liver metastasis from colorectal cancer patients. **(B) **Dicer mRNA level in normal mucosa, primary tumour and liver metastasis from colon cancer patients. **(C) **Dicer mRNA level in normal mucosa, primary tumour and liver metastasis from rectal cancer patients.

The mRNA level of Dicer between primary CRCs (2.863 ± 1.137) and the corresponding normal mucosa (2.851 ± 1.040) from 162 CRC patients did not show a significant difference (P = 0.835), while Dicer mRNA in liver metastasis (1.839 ± 1.148) was obviously lower than that in either the normal mucosa or the primary tumour (P < 0.0001, Figure 1A).

Further analyses on Dicer expression were done in comparison to the tumour site. In the colon (Figure 1B), Dicer was still not significantly different between normal mucosa (2.870 ± 1.066) and primary tumour (2.702 ± 1.204, P = 0.250) while the Dicer mRNA expression in liver metastases (2.081 ± 1.207) was significantly decreased in comparison to that in either normal mucosa (P = 0.003) or primary tumours (P = 0.034). In the rectum (Figure 1C), Dicer level in primary tumours (3.146 ± 0.952) was significantly increased compared to that in normal mucosa (2.816 ± 1.009, P = 0.034). Dicer in liver metastases (1.499 ± 0.999) was decreased in comparison to either the normal mucosa (P < 0.0001) or the primary tumour (P < 0.0001).

### Dicer mRNA level in relation to patient survival and other clinicopathological variables

**Figure 2:**
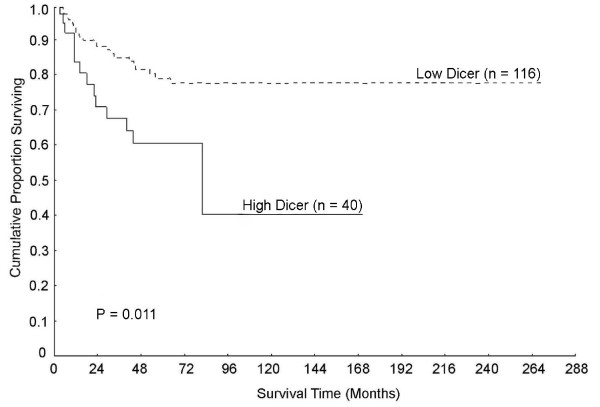
**A high level of Dicer in normal mucosa was connected to poor patient survival**.

We first focused on the mRNA level of Dicer in normal mucosa and found that a high level of Dicer in normal mucosa was related to poor survival (P = 0.011, Figure 2). Even in multivariate analysis including gender, age, tumour site, stage and differentiation, the relationship between Dicer and survival still remained significant (P = 0.0006, RR 3.682, 95% CI 1.749 - 7.750, Table 1). We also examined the survival significance of Dicer expression level in normal mucosa in a multivariate model including gender, age and tumour site (Table 1), as well as stage (non-lymph node metastasis vs. metastasis) and histological type (non-mucinous vs. mucinous carcinoma), the increased Dicer level was still significantly related to poor survival (P = 0.002, RR 3.447, 95% CI 1.588 - 7.481). There were no significant relationships between Dicer expression in normal mucosa and other clinicopathological variables including gender, age, tumour site, stage, differentiation and histological type (P > 0.05, data not shown).

**Table 1:** Multivariate analysis of the Dicer mRNA in normal mucosa, gender, age, tumour site, stage and differentiation in relation to patient survival

Variables	n	Hazard Ratio	95% CI	P
Dicer mRNA				0.0006
Low	106	1.000	-	
High	37	3.682	1.749 - 7.750	
Gender				0.462
Male	91	1.000	-	
Female	52	0.744	0.337 - 1.639	
				
Age (years)				0.052
≤ 72	60	1.000	-	
> 72	83	2.082	0.993 - 4.363	
Tumour site				0.072
Colon	88	1.000	-	
Rectum	55	0.478	0.214 - 1.069	
Stage				< 0.0001
I	20	1.000	-	
II	75	0.903	0.189 - 4.317	
III	33	3.651	0.793 - 16.803	
IV	15	16.726	3.335 - 83.871	
Differentiation				0.415
Better	103	1.000	-	
Worse	40	1.398	0.625 - 3.130	

**Figure 3:**
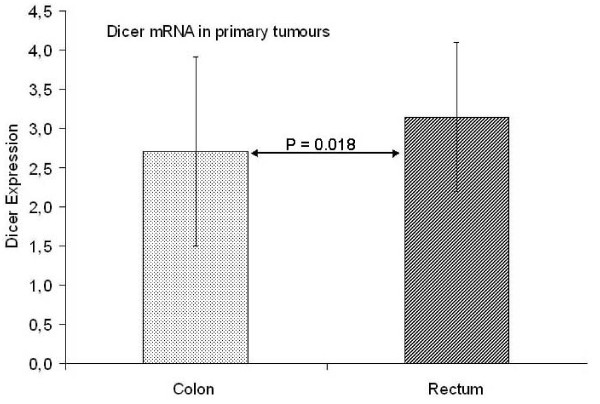
**Dicer expression in rectal cancer was higher than that in colon cancer**.

We then examined the Dicer mRNA level in primary tumours in relation to clinicopathological variables. There was no significant relationship between Dicer and survival in primary tumours. Focus on the tumour site showed that Dicer was significantly higher in rectal cancer (3.146 ± 0.953) than in colon cancer (2.703 ± 1.204, P = 0.018, Figure 3). There was no further significant relationship of Dicer to the other clinicopathological variables (P > 0.05, data not shown).

Finally, we examined Dicer in liver metastases in relation to clinicopathological variables. Liver metastasis samples were separated according to the size of the largest metastasis. It seems that the Dicer expression in smaller sized tumours (≤ 42.5 mm) was higher (2.068 ± 1.294) than in larger sized tumours (> 42.5 mm) (1.423 ± 0.622, P = 0.104). Patients with no metastases to other organs than the liver, showed a higher value of Dicer expression (1.948 ± 1.138) compared to distant metastases (1.447 ± 0.891) although the difference was not statistically significant (P = 0.158). When the patients were separated based on other clinicopathological variables including gender, age, the number of liver metastases, pre-chemotherapy, local recurrence and the time period for occurrence of liver metastases after primary tumour diagnosis, no significant relationship to Dicer expression was observed (P > 0.05, data not shown).

### Dicer mRNA in relation to miRNA expression in liver metastasis

Correlation studies between Dicer and miR-141, miR-200a, miR-200b, miR-200c, miR-429 expression in liver metastases revealed a positive correlation between Dicer mRNA and miR-141 expression (Pearson correlation coefficient, R = 0.419, P = 0.015, Table 2). There was also a positive trend between Dicer mRNA and miR-429 (R = 0.312, P = 0.077, Table 2). There was no significant relationship between Dicer level and the other miRNAs (P > 0.05, Table 2).

**Table 2:** Correlations between Dicer mRNA level and the miRNA expression in liver metastases

miRNA	R*	P
miR-141	0.419	0.015
miR-200a	0.239	0.180
miR-200b	0.110	0.543
miR-200c	- 0.132	0.463
miR-429	0.312	0.077

*Pearson correlation coefficient

## Discussion

In the present study, we examined Dicer mRNA level in the matched normal mucosa and primary tumour from 162 CRC patients and 37 liver metastases. We found a significant increase of Dicer mRNA expression in primary tumour from rectal cancer patients in comparison to that in the normal mucosa but there was no such evidence in colon cancer patients, suggesting that Dicer may play a different role in the development of rectal cancer from its role in colon cancer. This assumption led to a direct comparison of the Dicer mRNA level in primary tumours from rectal and colon cancer, which proved that the Dicer mRNA level was significantly up-regulated in rectal cancer compared to colon cancer, indicating that Dicer was differently regulated in the two sites. In earlier studies where CRC was divided into different sub-types according to the site where the cancer occurred, it was shown that different types of this cancer exhibited their own specific genetic features like microsatellite instability in proximal colon cancer whereas distal colon cancers showed chromosome instability [25]. Even though rectal cancer is similar to colon cancer it exhibits its own specific features, for instance, up-regulated nuclear *b-catenin *[26], as well as a higher level *of Cox-2 *than in colon cancer [27]. *K-ras *mutations are more common in colon cancer than in rectal cancer, the number of mutations in colon tumours is higher than in rectal tumours, and the mutational pattern restricted to the *APC *gene is more frequent in rectal than colon cancer [28]. Therefore our results may provide further support that CRC could be differentiated into two different diseases, colon cancer and rectal cancer.

Comparison of the Dicer mRNA level in liver metastases to that in normal mucosa and primary tumours from colon and rectal cancer exhibited a significant decrease of Dicer mRNA level in liver metastases. Similar results were obtained in breast cancer, where Dicer was decreased in metastases due to inhibition by specific miRNAs [9]. Our assumption gets further support from two studies in ovarian cancer patients, which indicated that a reduced Dicer mRNA level is associated with advanced tumour stage [12,16]. Similar results were seen in 67 NSCLCs, where a reduced Dicer expression level was found in poorly differentiated tumours [14].

Interestingly, in the present study, higher Dicer expression in normal mucosa was related to a worse survival independently of gender, age, tumour site, stage and differentiation. Dicer up-regulation was found in prostate cancer, where an increased Dicer level was significantly associated with aggressive cancer features. This up-regulation in prostate cancer was suggested to be induced by a genomic instability at chromosome 14 (14q32) due to amplification [18]. It is known that normal mucosa, which is assumed to be uninvolved during cancer formation and apparently normal at the morphological level, shows abnormal gene regulation in comparison to normal mucosa from non-cancer patients [29]. Dicer involvement in early cancer development has been reported in lung cancer, where precursor lesions of lung adenocarcinoma i.e. adenomatous hyperplasia, showed Dicer over-expression [13]. These findings would lead to the conclusion that the higher Dicer mRNA level in normal mucosa has a certain role in early cancer formation.

Examination of Dicer mRNA level in liver metastases in association with clinicopathological variables was not statistically relevant in the tested variables (largest tumour diameter, distant metastasis excluding the liver, gender, age, number of liver metastasis, pre-chemotherapy, local recurrence and period of time until occurrence of liver metastasis after primary tumour diagnosis). This finding was also reported in breast cancer patients where low or high Dicer mRNA levels were not significantly associated with metastases or patient outcome [9]. Nonetheless, it seemed that metastases with a larger size showed a lower Dicer mRNA level than metastases with a smaller size. Far distant metastases excluding liver metastases showed a lower Dicer expression than those in the liver. These results suggest that a low Dicer mRNA level in metastases might be involved in facilitating the metastatic spread for distant metastases as was presumed in breast cancer [15].

Our miRNA correlation study showed that the Dicer mRNA level was positively associated with miR-141 in liver metastasis samples. The miR-141 is known to be mainly involved in activation of epithelial differentiation in breast, pancreatic and CRC. A decreased miR-141 level promotes the drift into metastatic behaviour of cancer cells, due to repression of miR-141 by ZEB1 [30]. However our results, showing four out of five tested miRNAs were not correlated to Dicer support the fact that more factors are involved in the aberrant levels of miRNAs during cancer progression.

How a decreased Dicer level was associated with the change from primary to metastatic tumour in CRC, as well as in relation to miRNAs and clinicopathological variables, needs to be confirmed in a larger cohort of patients. Our miRNA study in liver metastasis was a pilot study to gain first insights into how miRNA regulation might be connected to Dicer in liver metastasis from CRC patients. Since the liver metastases were independent from the matched normal and primary cancer tissue, the results of comparison of the Dicer expression in liver metastasis with primary tumour/normal mucosa was a weakness. In addition analysis of global tumour RNA levels may miss subtleties of tissue expression that are crucial for tumour behaviour. The expression of mRNA in a tissue fragment may not necessarily equate with the mRNA expression by the tumour.

## Conclusion

Dicer is up-regulated in the earlier development of rectal cancers, and the increased Dicer expression in normal mucosa is an independent prognostic factor in CRC patients. These findings strengthen the role of Dicer in making early diagnosis, evaluating survival and planning therapy of CRC patients.

## Competing interests

The authors declare that they have no competing interests.

## Authors' contributions

JS carried out the main experiments and drafted the manuscript. CJW participated in the project design, valuable discussion and statistical analysis. SG participated in the real-time PCR experimental procedure with primary colorectal cancer and liver metastasis samples. ÅW did the experiment on liver metastases. DH revised and helped in drafting the manuscript. XFS and HZ conceived of the study, participated in the design and helped drafting the manuscript. All authors read and approved the final manuscript.

## Pre-publication history

The pre-publication history for this paper can be accessed here:

http://www.biomedcentral.com/1471-2407/11/345/prepub
